# Perinatal arterial ischemic stroke: how informative is the placenta?

**DOI:** 10.1007/s00428-024-03780-1

**Published:** 2024-03-19

**Authors:** Jessica Hirschel, Francisca Barcos-Munoz, François Chalard, Florence Chiodini, Manuella Epiney, Joel Fluss, Anne-Laure Rougemont

**Affiliations:** 1 Division of Neonatal and Intensive Care, Department of Pediatrics, Gynecology and Obstetrics, University Hospitals of Geneva, Geneva, Switzerland; 2 Unit of Pediatric Radiology, Department of Radiology, University Hospitals of Geneva and Faculty of Medicine, University of Geneva, Geneva, Switzerland; 3 Therapeutic Tissue Biobank, University Hospitals of Geneva, Geneva, Switzerland; 4 Obstetrics Unit Department of Obstetrics and Gynecology, University Hospitals of Geneva and Faculty of Medicine, University of Geneva, Geneva, Switzerland; 5 Pediatric Neurology Unit, University Hospitals of Geneva and Faculty of Medicine, University of Geneva, Geneva, Switzerland; 6 Division of Clinical Pathology, Diagnostic Department, Geneva University Hospitals and Faculty of Medicine, University of Geneva, Geneva, Switzerland

**Keywords:** PAIS, Neuroplacentology, Amsterdam consensus, Crack cocaine, Drug abuse, SARS-CoV-2

## Abstract

Neuroplacentology is an expanding field of interest that addresses the placental influence on fetal and neonatal brain lesions and on further neurodevelopment. The objective of this study was to clarify the link between placental pathology and perinatal arterial ischemic stroke (PAIS). Prior publications have reported different types of perinatal stroke with diverse methodologies precluding firm conclusions. We report here the histological placental findings in a series of 16 neonates with radiologically confirmed PAIS. Findings were grouped into 3 categories of lesions: (1) inflammation, (2) placental and fetal hypoxic lesions, and (3) placentas with a high birthweight/placenta weight ratio. Matched control placentas were compared to the pathological placentas when feasible. The eight term singleton placentas were compared to a series of 20 placentas from a highly controlled amniotic membrane donation program; in three twin pregnancies, the placental portions from the affected twin and unaffected co-twin were compared. Slightly more than half (9/16, 56%) had histopathological features belonging to more than one category, a feature shared by the singleton control placentas (13/20, 65%). More severe and extensive lesions were however observed in the pathological placentas. One case occurring in the context of SARS-CoV-2 placentitis further expands the spectrum of COVID-related perinatal disease. Our study supports the assumption that PAIS can result from various combinations and interplay of maternal and fetal factors and confirms the value of placenta examination. Yet, placental findings must be interpreted with caution given their prevalence in well-designed controls.

**Supplementary Information:**

The online version contains supplementary material available at 10.1007/s00428-024-03780-1.

## Introduction

In eutherian mammals, the placenta plays a crucial role in the development and survival of the fetus, extending beyond pure fetal development, with the placenta influencing several aspects of a human life into adulthood. The placenta, a key player in fetal programming, has been linked in particular to cardiovascular comorbidities [1], psychiatric disorders such as autism [2], and neurological morbidity [3]. The placental influence on the brain, also known as neuroplacentology, is a growing field of interest [3]. In neonatal hypoxic-ischemic encephalopathy, and specifically in term newborns, histological examination of the placenta may identify clinically overt or silent, preexisting antepartum pathophysiologic processes that may directly cause CNS damage, decrease the threshold for neurologic injury, or serve as markers for a deleterious in utero environment [4, 5]. The role of the placenta is of particular interest in perinatal arterial ischemic stroke (PAIS). PAIS generally remains an isolated event as opposed to stroke occurring later in life, and may be due to local intracranial vascular pathologies, or result from distant embolism from another site such as extracranial vessels, the heart, the umbilical vein, or more likely, from the placenta. Converging evidence identifies the placenta as the main plausible source of emboli to the fetal brain circulation [6, 7], but studies on placenta histopathology in PAIS have been limited for several reasons. First, although the adverse events leading to PAIS occur shortly before birth, the neonate is often asymptomatic at birth which delays diagnosis; the placenta may by then have been discarded. Second, lack of standardization in placental pathology reporting has led to discrepancies between studies. Third, finding appropriate controls is challenging. Control placentas are biased by the pathologies or obstetrical events motivating their analysis, and case–control studies are rare.

In this study, we address the role of placental pathology in a series of 16 neonates diagnosed with PAIS. Histological findings were described according to the Amsterdam consensus terminology [4]. Placenta lesions allowed for classification into 3 distinct categories: (1) inflammation, (2) placental and fetal hypoxic lesions, and (3) placentas with a high birthweight/placenta weight ratio. Whenever feasible, we provide comparison with strictly defined controls.

## Materials and methods

### Patient selection and characteristics

Infants born in the University Hospital of Geneva over a period of 7 years and 5 months, between October 2016 and March 2023, with a diagnosis of PAIS and whose placentas were available for analysis were enrolled.

Data was retrospectively retrieved from the Swiss Neuropediatric Stroke Registry (SNPSR) for children born before June 2018, while infants born thereafter were prospectively included after parental consent. The SNPSR registry enables access to maternal, fetal, and neonatal data.

PAIS was defined as stroke occurring between the 20th gestational week and the 28th postnatal day of life, with evidence for focal arterial ischemic stroke on a brain MRI performed between days 1 and 25 of life (mean 8.2, median 4). Imaging was performed using a 1.5-T Avanto MRI scanner (Siemens, Erlangen, Germany). No sedation was required. Each MRI protocol included at least the following sequences: 3D T1 mprage, coronal T2 TSE, axial T1 IR, axial susceptibility-weighted image (SWI), and axial diffusion-weighted imaging (DWI) with apparent diffusion coefficient (ADC) map. MRI data were all reassessed by an experienced pediatric neonatal stroke specialist (JF) and a pediatric radiologist (FC) to reach agreement on the nature and localization of the stroke. Lesions were classified according to the classification of perinatal arterial ischemic stroke territory subtypes proposed by Wagenaar et al. [8].

### Placenta histology and immunohistochemistry

Placentas were processed according to standard protocol and consensus guidelines [4]. Placental weight was recorded on fresh specimens, after trimming of the extraplacental membranes and umbilical cord. Representative sections and pathological findings from the cord, membranes, and placental disk were sampled. For routine histological examination, 3-μm-thick sections were stained with hematoxylin–eosin (H&E). For immunohistochemistry, SARS-CoV-2 nucleocapsid detection was performed using the Bio SB SARS-CoV-2 BSB-134 clone (Mouse Monoclonal, dilution 1:100).

### Pathology criteria

Ascending intrauterine infection, villitis of unknown etiology (VUE), chronic deciduitis, maternal vascular malperfusion (MVM), and fetal vascular malperfusion (FVM) were reported and graded according to the Amsterdam consensus statement [4, 9]. Chorangiosis was defined as ≥ 10 capillaries in ≥ 10 terminal villi in ≥ 10 areas of the placenta using a × 10 objective [10]. Increased syncytial knots was reported when more than 33% of the villi showed knots [4]. Massive perivillous fibrin deposition (MPFD) was defined as perivillous fibrinoid material encasing at least 50% of the villi, extending from the maternal to the fetal surface, on at least one slide [11]. Coexistence of MPFD and trophoblast necrosis with identification of SARS-CoV-2 viral protein in placental tissue was considered as placental destruction and insufficiency from COVID-19 [12, 13].

### Classification of the histopathological findings in 3 categories of lesions

We chose to group the findings into 3 distinct categories of lesions:

1. Inflammation
2. Placental and fetal hypoxic lesions
3. Placentas with a high birthweight/placenta weight ratio (BW/PW ratio)

*Category 1* includes ascending intrauterine infection, VUE, and chronic deciduitis.

*Category 2* includes lesions resulting from oxidative stress and hypoxia to the placenta that may originate from the maternal circulation (i.e., MVM) or the fetal circulation (i.e., FVM) [14]. Since non-perfused/avascular villi encased in MPFD result in impaired fetoplacental circulation and fetal hypoxic-ischemic injury, SARS-CoV-2 placentitis was considered within the hypoxemic category [15]. Villous stromal hemorrhage was indicative of acute hypoxemia. Meconium staining of the membranes and increased circulating nucleated red blood cells (NRBCs) were markers of fetal hypoxia. Finally, chorangiosis and increased extravillous trophoblast (EVT) were considered as adaptive mechanisms to prolonged chronic hypoxia.

*Category 3* corresponds to placentas that are small relative to neonatal birthweight.

### Selection of controls

Defining “control placentas” is a complex and subjective task. We chose a stringent approach, allowing for the selection of 2 categories of controls:

1. The singleton term placentas from our series were compared to placentas from an amniotic membrane donation program. These control placentas were from highly selected term pregnancies, with no obstetrical or maternal complications. Neonates displayed no neurological symptoms.
2. For twin pregnancies, the placental disk/portion from the unaffected co-twin was used as control.

## Results

### Clinical characteristics of PAIS cases and controls

Sixteen neonates diagnosed with PAIS were included, 9 males and 7 females. Thirteen were born from singleton pregnancies. Of the three twin pregnancies, only one was spontaneous, the other two pregnancies following oocyte donation, or in vitro fertilization (IVF). Gestational age ranged between 31 + 2 gestational weeks (GW) and 41 + 5 GW, with 2 early preterm births, 5 preterm births, and 9 full-term deliveries. The majority of neonates (12/16) developed neurological symptoms between days 1 and 3 of life; the remaining 4 newborns were diagnosed following brain ultrasound routinely performed in the setting of prematurity.

The clinical characteristics of the PAIS cases and of the controls can be found in supplemental Table S1.

### Placenta histopathological findings, neonates with PAIS

**Fig. 1:**
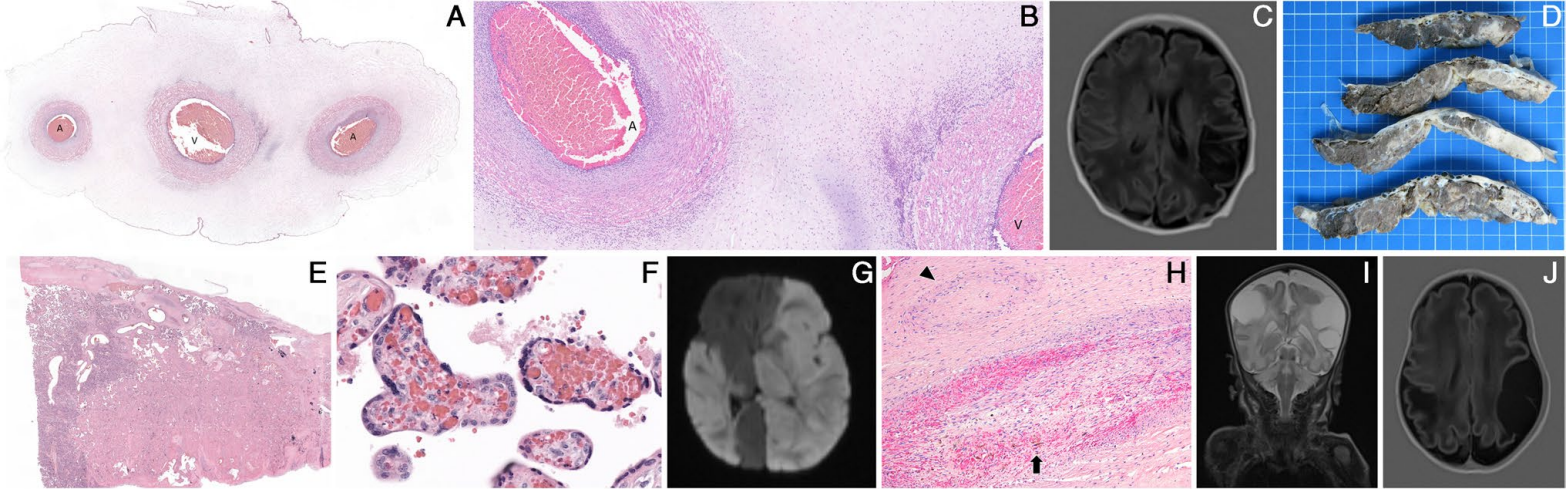
Illustrative gross and microscopic placenta pathological findings of the main categories of lesions. Associated brain MRI findings are shown. Case P2: inflammation. Ascending intrauterine infection, premature delivery at 33 + 6 GW. **A**, **B** Umbilical cord phlebitis (*V*, umbilical vein) and arteritis (*A*, umbilical arteries) identify a stage 2, grade 2 fetal inflammatory response (hematoxylin & eosin, H&E, **A** × 10, **B** × 60). **C** Left posterior MCA old infarct seen on axial T1 MRI at day 15. Case P6: hypoxia and high BW/PW. PW < P10, BWP50–P75, and term delivery. **D**, **E** On cut section, 20% of the placenta approximately showed large firm whitish areas (**D**), corresponding histologically to maternal vascular malperfusion (MVM) and large areas of infarction (**E**, H&E whole-mount view). Areas of villous stromal hemorrhage were consistent with acute hypoxia (**F**, H&E × 400). **G** Imaging findings at day 1 demonstrate on DWI massive acute left hemispheric stroke and right MCA stroke. Case P14: hypoxia. Diamniotic-dichorionic twin pregnancy, early premature delivery at 31 + 2 GW. **H** FVM was seen in the affected twin only. Villous stromal-vascular karyorrhexis, with iron deposition (*arrow*), and stem vessel obliteration (*arrowhead*) are shown. **I**, **J** Imaging findings (coronal T2 and axial T1) at term demonstrate bilateral porencephalic cavities in the MCA territories, more extensive on the left side

The histopathological features of the 16 placentas are summarized in Table 1. Slightly more than half the placentas (9/16, 56%) showed features belonging to more than one category. Representative examples from the 3 categories of lesions are illustrated in Fig. 1.

**Table 1:**
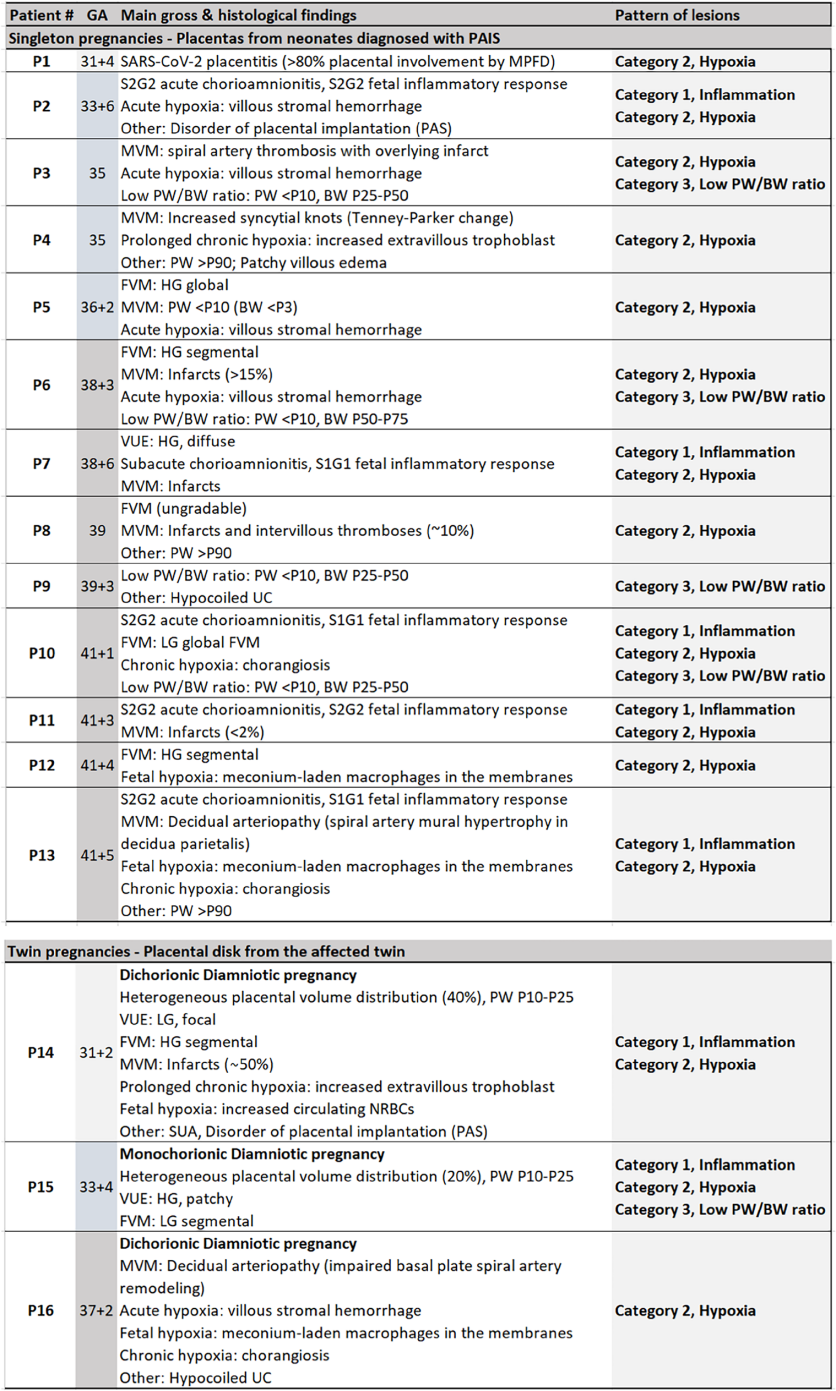
Main histological findings, placentas from neonates diagnosed with PAIS

*PAIS*, perinatal arterial ischemic stroke; *GA*, gestational age; *PW*, placenta weight; *BW*, birth weight; *UC*, umbilical cord; *S*, stage; *G*, grade; *HG*, high grade; *LG*, low grade; *MVM*, maternal vascular malperfusion; *FVM*, fetal vascular malperfusion; *MFPD*, massive perivillous fibrin deposition; *VUE*, villitis of unknown etiology; *light grey*, early preterm delivery; *light blue*, preterm delivery; *darker grey*, full-term pregnancy

#### Category 1: inflammation

Evidence of ascending intrauterine infection was seen in 5 singleton placentas (5/16, 31%), one preterm, the other full term, that also displayed histological signs of hypoxia (Category 2 lesions). Two cases showed a S2, G2 fetal inflammatory response, i.e., severe acute umbilical phlebitis and arteritis were seen. The other three placentas showed acute non-severe S1, G1 umbilical phlebitis. One case also displayed high-grade, diffuse VUE.

VUE was seen in two twin placentas.

#### Category 2: placental and fetal hypoxic lesions

Features consistent with placental and fetal hypoxia were the most frequent findings, (15/16, 94%), consisting mainly of FVM and MVM.

FVM, identified in 7 placentas (7/16, 44%), was high grade in 4 cases, low grade in 2 placentas, and ungradable in the last (Case P8) due to freezing artifacts. The intensity of the lesions was therefore variable, ranging from recent intramural fibrin deposition in stem vessels, to high grade FVM with multiple chorionic plate and stem vessel thromboses, villous stromal-vascular karyorrhexis, and large areas of avascular villi. In one placenta (P12), focal vessel wall calcification was seen, together with umbilical vein, chorionic plate vessel, and stem vessel thrombi. High grade FVM was associated in two cases with signs of an acute hypoxic event to the placenta [16] (villous stromal hemorrhage) and with meconium-laden macrophages in the membranes or increased NRBCs and EVT in one case each. Meconium-laden macrophages in the membrane chorion have been associated with an increased likelihood of oropharyngeal meconium and increased risk of meconium aspiration [17]. Of note, FVM may have been overlooked in the sole placenta without histological evidence for hypoxia (P9), since evaluation was limited by freezing artifacts, the placenta having been sent with delay to the pathology ward. In 4 of the 7 cases with FVM, features of MVM were associated.

MVM without FVM was seen in 6 further cases. Two cases showed associated signs of acute or prolonged chronic hypoxia. Meconium staining of the membranes was also seen.

There is no consensus on the definition of high grade MVM. Provisional criteria consist of a placental weight < 3rd centile with at least one of the following: accelerated villous maturation, distal villous hyperplasia, and multiple infarcts [18]. One preterm singleton placenta (P5) met these criteria.

**Fig. 2:**
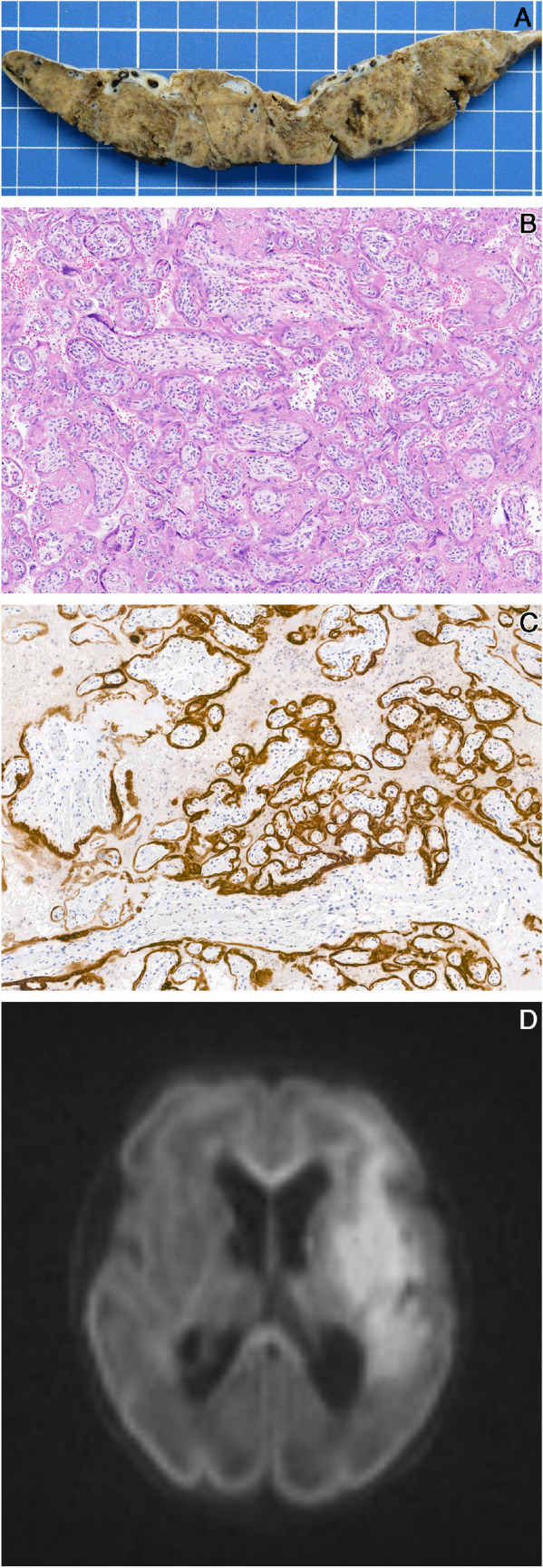
Case P1: SARS-CoV-2 infection. **A** Macroscopic placenta section, early preterm delivery (31 + 4 GW), showing extensive fibrin deposition, involving > 80% of the total placenta volume. **B** Histology showed partially necrotic chorionic villi encased in fibrin (hematoxylin & eosin, H&E, × 100). **C** Circumferential cytotrophoblastic and syncytiotrophoblastic reactivity to SARS-CoV-2 nucleocapsid antibody (× 100). **D** Imaging at day 3 shows acute left MCA infarct on DWI

The one case of SARS-CoV-2-related MPFD showed extensive parenchymal damage, involving more than 80% of the placenta volume, expected to result in fetal hypoxia (P1, Fig. 2). There was no evidence of coexisting chronic histiocytic intervillositis.

**Fig. 3:**
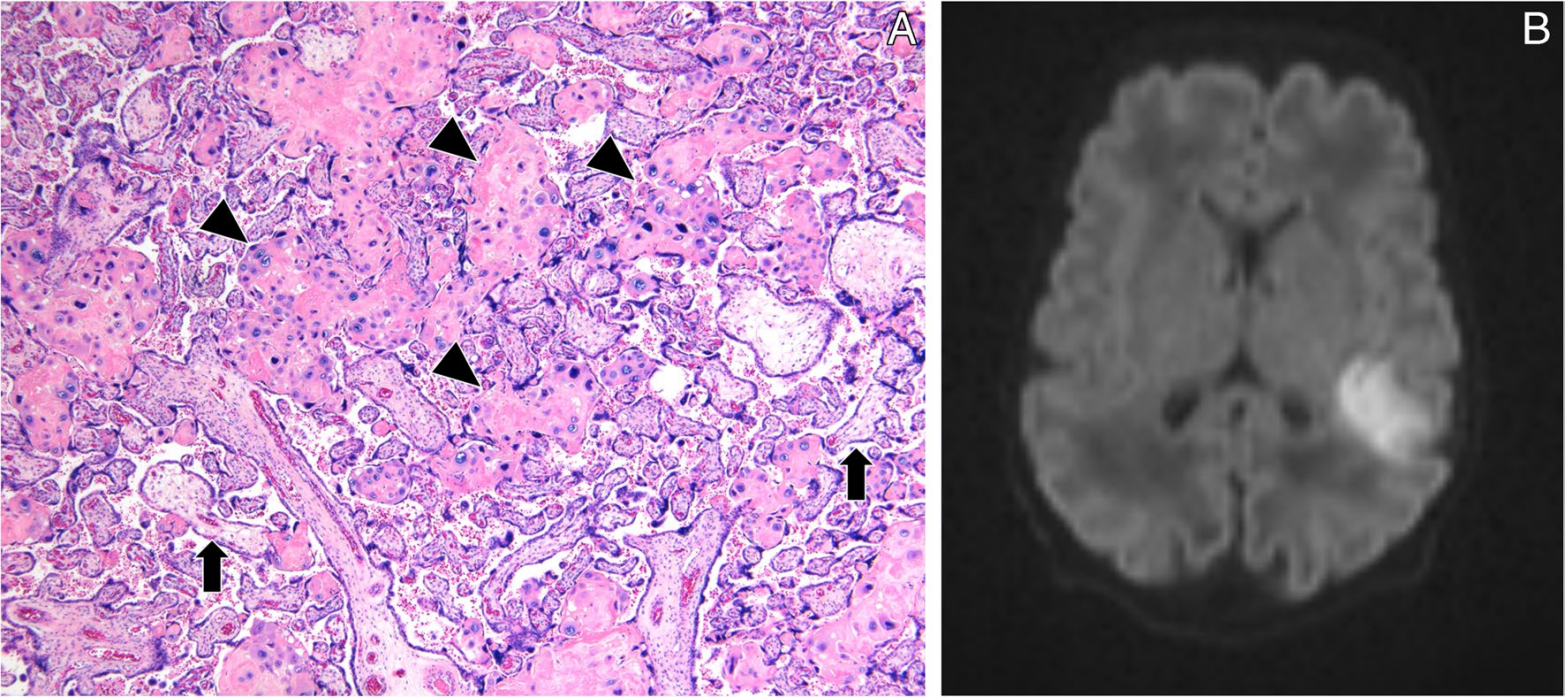
Case P4: in utero crack cocaine exposure. **A** Increased extravillous trophoblast with atypia is seen (*arrowheads*), together with focal villous edema (*arrows*). **B** DWI MRI at day 5 demonstrates acute superficial left posterior MCA stroke

Finally, excessive EVT and increased syncytial knots consistent with chronic hypoxia, and patchy villous edema were documented in a placenta from a polydrug user (P4, Fig. 3), with prenatal exposure of the neonate.

#### Category 3: placentas with a high BW/PW ratio

Five placentas, one twin and four singleton, were considered small relative to neonatal birthweight. Except for the one placenta with freezing artifact discussed above (P9), all showed features of hypoxia (Category 2), and three also displayed inflammation (Category 3).

### Comparison with controls

#### Term pathological placentas vs. normal term controls from the membrane donation program

Similar to the PAIS placentas, lesions of more than one category were seen in a majority of the control cases (13/20, 65%).

All 20 control placentas showed some degree of hypoxic lesions. MVM was seen in 13 cases (13/20, 65%), and FVM in 9 (9/20, 45%); there was no high grade MVM or FVM. Meconium staining of the membranes was frequent (9/20, 45%). Inflammation was observed in 11 placentas (55%). Ascending intrauterine infection was mild only (S1, G1 maternal/fetal response). Two cases showed high-grade, patchy VUE and five chronic deciduitis (of which only one also had VUE).

Finally, high BW/PW ratio (Category 3 lesions) was seen in 4 cases.

Severe/high grade lesions were seen in the PAIS placentas only, with the exception of high-grade VUE that was seen in both PAIS and control placentas. Statistical significance (*p* = 0.0398) was reached for severe ascending intrauterine infection, defined as S3 and/or G2 maternal and/or fetal inflammatory response.

#### Twin placentas: affected twin vs. co-twin

In all three twin pregnancies, one twin developed neonatal stroke, while the co-twin remained unaffected. Signs of hypoxia were seen in both disks/portions from the affected, and unaffected co-twin, but no co-twin portion showed histological features from more than one category. Differences between the two placental disks/portions are reported in detail below.

#### Placenta #14: early preterm dichorionic-diamniotic twin placenta

The disk from the affected twin showed extensive hypoxic lesions, with high grade FVM, and severe MVM with infarcts, affecting approximately half the placenta disk volume, together with signs of prolonged chronic hypoxia. There were mild differences in relative placental volume, the affected twin receiving 40% of the total volume. Finally, low-grade focal VUE was seen in the affected twin disk only.

#### Placenta #15: preterm monochorionic-diamniotic twin placenta

The placental portion from the affected twin showed features from all three categories. Volume distribution was highly heterogeneous, with only 20% of the volume attributed to the affected twin. High-grade, patchy VUE was seen in this portion only, as well as low grade FVM. The much larger placental volume devoted to the unaffected twin showed only minor hypoxic features, with peripheral infarcts representing < 2% of the volume (in a preterm placenta however).

#### Placenta #16: term fused dichorionic-diamniotic twin placenta

The two disks showed chorangiosis (adaptation mechanism) and signs of acute hypoxia. The disk from the affected twin showed additional hypoxic features, meconium staining of the membranes, and decidual arteriopathy.

A comparison of the prevalence of severe or high grade lesions between affected neonates and controls is provided in Table 2. The detailed histological features of the control placentas can be found in Supplemental Tables S2 and S3.

**Table 2:** Severe/high grade lesions: comparison between placentas from affected neonates and controls

Singleton pregnancies			
	Term PAIS neonates (*n* = 8)	AMD controls (*n* = 20)	OR/*p* value
Category 1, inflammation			
Severe ascending intrauterine infection	3/8	0/20	**0.0398**
HG VUE	1/8	2/20	0.8471
Category 2, hypoxia			
High-grade FVM	2/8	0/20	0.1201
High-grade MVM	0/8	0/20	‒
Twin pregnancies			
	Affected twin (*n* = 3)	Unaffected co-twin (*n* = 3)	OR/*p* value
Category 1, inflammation			
Severe ascending intrauterine infection	0/3	0/3	‒
HG VUE	1/3	0/3	0.5415
Category 2, hypoxia			
HG FVM	1/3	0/3	0.5415
HG MVM	0/3	0/3	‒

*HG*, high grade; *LG*, low grade; *MVM*, maternal vascular malperfusion; *FVM*, fetal vascular malperfusion; *VUE*, villitis of unknown etiology

Severe ascending intrauterine infection: defined as stage 3 and/or grade 2 maternal and/or fetal response

## Discussion

Given the nature of our cohort, we will first summarize the general findings, before discussing the conclusions drawn by the case–control groups. Then, we will provide a more in-depth discussion of two cases of particular interest, one with diffuse placental damage due to SARS-CoV-2 infection, the other with chronic hypoxic lesions likely secondary to polydrug abuse.

Hypoxic lesions were as expected the most frequent lesions in PAIS placentas (94%), followed by inflammation (44%). A majority of the PAIS placentas presented concomitant lesions from different categories (56%). Placentas from affected singleton neonates tended to show more severe lesions than controls. Lesions were also more numerous on the affected twin side.

Recent studies have linked several maternal and fetal risk factors to PAIS such as chorioamnionitis or small for gestational age (SGA), suggesting a multifactorial pathogenesis [19–21]. This is reflected by different histological findings in our series, which confirm the previous results showing that multiple lesions tend to coexist in neuroplacentology [22]. Multiple lesions were documented not only in our PAIS series but also surprisingly in the control placentas that frequently also displayed lesions belonging to more than one of the three categories (7/13 PAIS singleton placentas, 53%; vs. 13/20 singleton controls, 65%). Multiple patterns of lesions were described in 38.7% of term pregnancies with normal outcome reported by Romero et al. [23]. These results highlight the interpretation complexities, given the presence of some form of placental lesions even in highly controlled pregnancies with no delivery or neonatal complications. However, most lesions in control placentas were mild.

Fetal vascular malperfusion is considered as fetal blood flow obstruction, comprising a group of lesions attributed to absent or reduced perfusion of the villi [9]. FVM has been associated with an increased risk of neonatal encephalopathy and neonatal stroke [24]. FVM was seen in more than one-third of the PAIS placentas and surprisingly in 44% of the controls. The finding of two or more FVM lesions in a placenta is uncommon (< 1%) in normal pregnancies [23]. Although MVM was the most frequent observation, lesion impact was highly variable, ranging from small peripheral infarcts in a term placenta of minor if any significance, to infarcts involving more than half the placenta volume. High-grade or diffuse FVM or MVM lesions were observed in PAIS placentas only. Correlation between adverse clinical outcome and pathological findings is obviously expected to be higher with diffuse and high grade lesions, but other factors creating a favorable context for stroke are also probably at play. Histological features of FVM (thromboses of varying ages, avascular villi…) suggest that lesions evolve during a subacute to chronic period before birth. MVM lesions also exemplified maternal underperfusion manifesting at different time points, both of abrupt onset (infarcts) and gradual onset, of intermediate (increased syncytial knots) or prolonged duration (high BW/PW ratio) [25]. Clinical and imaging findings support the occurrence of PAIS close to the time of birth, including those diagnosed later in infancy. Impaired fetal blood flow and oxygenation secondary to FVM has been postulated to prime the brain to acute injury in the per partum period in conditions that would otherwise have no consequences [26]. A stroke could therefore occur in a neonate with an unfavorable environment, “weakened” by chronic stressors and a pathological placenta, thereby imparting an “acute upon chronic” pattern of lesions [26, 27].

Inflammation was seen in 7 PAIS placentas, of which 3 also had clinical chorioamnionitis. Interestingly, although 7 singleton term control placentas had signs of acute inflammation after elective cesarean section, the maternal and fetal inflammatory responses were never more severe than S1G1. This is in accordance with studies describing the common occurrence of mild acute inflammation (grade 1, stage 1–2, fetal, and maternal inflammatory responses) in normal deliveries [28]. Conversely, umbilical cord arteritis was seen in PAIS placentas only. Involvement of the umbilical arteries (Stage 2 fetal inflammatory response) has been linked to higher rates of adverse neonatal outcome due to higher plasma concentrations of interleukin-6 [29]. In our series, differences in the prevalence of severe ascending intrauterine infection between term singleton PAIS and control placentas reached statistical significance. VUE was seen in both the pathological placentas and the controls and chronic deciduitis in control placentas solely. Chronic placental inflammatory lesions were reported by Romero et al. in 29.9% uncomplicated pregnancies [23]. The association of chorioamnionitis with neonatal brain lesions including stroke is well established [30, 31], but pathogens are rarely identified despite the histopathological diagnosis of chorioamnionitis [22]. Cytokine elevation in inflammation or infection induces prothrombotic events such as endothelial activation, hypercoagulability, platelet activation, and impaired fibrinolysis. The close interaction between the inflammatory and coagulation cascades suggests that inflammation may promote thrombus formation in the placenta, umbilical cord, or other vessels supplying the brain. Cerebral arteries susceptible to stroke show a higher expression of proinflammatory cytokines compared to non-susceptible arteries [32]. Focal arteritis and thrombosis caused by susceptibility to proinflammatory mechanisms may therefore contribute to the inflammatory pathophysiology of stroke.

In the twin cases, more severe and numerous lesions were found on the placental portion of the affected twin. Cases P14 and P15 clearly showed reduced effective placental volume and higher degrees of hypoxic lesions in the affected twin portion. In mild placental insufficiency, the brain’s ability to prioritize oxygen supply is effective in maintaining adequate oxygenation [33]. However, as the hypoxic condition persists, there is a point beyond which cerebral blood vessels no longer vasodilate as effectively, while the resistance to blood flow in the placenta continues to rise. The weight of the two affected twins was below the 10th centile. In severe intrauterine growth restriction (IUGR), the brain’s ability to prioritize oxygen supply eventually fails, leading to the significant clinical challenge of preventing hypoxic brain injury.

Why are certain territories more affected than others? One hypothesis would be that blood flow directed to areas of lower resistance would benefit some areas to the detriment of others.

The characteristics of the two cases illustrated in Figs. 2 and 3, both showing extensive placental involvement, need to be highlighted.

First, in Case P1, neonatal stoke occurred in the context of a SARS-CoV-2 infection, the mother having declined vaccination. COVID-19 is associated with adverse pregnancy outcomes and neonatal complications [34, 35]. SARS-CoV-2 placentitis is defined by the coexistence of 3 histological findings: (1) chronic histiocytic intervillositis, (2) increased fibrin deposition, and (3) trophoblast necrosis [15]. Although not specific to SARS-CoV-2 and of still unclear etiology, MPFD is associated with IUGR, preterm labor, and stillbirth [36, 37]. Severe placental SARS-CoV-2 infection can impact neonatal outcome even in the absence of vertical transmission [38]. Few case reports document severe neurological damage with cystic periventricular leukomalacia in the newborn of mothers with SARS-CoV-2 placentitis [39, 40], but to the best of our knowledge there has been no previous report of SARS-CoV-2 placentitis-associated PAIS.

Extensive and unusual histological findings attributed to the hypoxia category were observed in a premature neonate exposed in utero to cocaine, cannabis, and tobacco (P4). Fetal cocaine exposure has been linked to IUGR, stillbirth, preterm delivery, and placental abruption [41, 42]. The neurotoxic effects of cocaine are potentiated by pyrolysis and are therefore majored with exposure to the smoked form of cocaine (crack cocaine) [41]. Cocaine exerts major vasoconstrictive effects on the uterine vessels resulting in fetal hypoxia, with compensatory increases in blood flow to the fetal brain and heart [43]. Histology in documented cases of cocaine or crack abuse ranges from an absence of specific placental findings [44, 45] to chorionic villus edema and hemorrhage [46]. Hypovascularization of the villi has been potentially ascribed to uteroplacental vasoconstriction and chronic underperfusion [47]. Chronic exposure to tobacco in early pregnancy is also associated with hypoxia, resulting in reduced cytotrophoblastic invasion, and increased EVT islands [48]. The increase in EVT islands was particularly striking in this placenta from a polydrug user. Cells showed nuclear pleomorphism, with irregular and enlarged, hyperchromatic nuclei. These cells are known to become aneuploid from endoreduplication [49]. Pleomorphism is often seen in EVT; it is noteworthy however that ploidy changes may be promoted by hypoxia [50]. The associated patchy villous edema, sufficient to induce placental hypertrophy (placenta weight > P90), may represent further indication of microcirculatory anomalies with flow changes in the fetal circulation [51].

## Conclusion

Even though larger cohorts are needed to draw more definitive conclusions on the placental implications in neonatal stroke, our study brings insight into important aspects of the placenta-brain interaction. We describe the occurrence of PAIS in specific clinical contexts, namely, COVID-19 infection, drug abuse, and twin pregnancies. All stroke cases presented some form of placental lesions and compared to controls showed more severe lesions. On the other hand, the presence of significant lesions in healthy control pregnancies points towards an additional multifactorial background permissive to stroke. Multiple elements including perinatal risk factors appear to be implicated in PAIS outcome. Identifying those risk factors and understanding their implication on placental remodeling remains a challenge, which has to be addressed to design potential preventive measures.

Large-scale studies using consensus histology reporting [4, 9] will facilitate future comparisons; in the meantime, we can rely on observational studies to increase our understanding of neuroplacentology.

### Supplementary Information

Below is the link to the electronic supplementary material.

Supplementary file1 (XLSX 17 KB)

Supplementary file2 (XLSX 11 KB)

Supplementary file3 (XLSX 13 KB)

## Data Availability

All the data derived from this study are included in the manuscript. Further information can be made available by contacting the last author (anne-laure.rougemont@hug.ch).

## Abbreviations

ADC: Apparent diffusion coefficient
BW: Birthweight
CNS: Central nervous system
CS: Cesarean section
DWI: Diffusion-weighted imaging
EVT: Extravillous trophoblast
FVM: Fetal vascular malperfusion
G: Grade
GA: Gestational age
GW: Gestational weeks
H&E: Hematoxylin–eosin
HG: High grade
ICA: Internal carotid artery
IUGR: Intrauterine growth restriction
IVF: In vitro fertilization
LG: Low-grade
MAR: Medically assisted reproduction
MCA: Middle cerebral artery
MPFD: Massive perivillous fibrin deposition
MRI: Magnetic resonance imaging
MVM: Maternal vascular malperfusion
NRBCs: Nucleated red blood cells
PAIS: Perinatal arterial ischemic stroke
PAS: Placenta accreta spectrum
PROM: Premature rupture of membranes
PW: Placenta weight
S: Stage
SGA: Small for gestational age
SWI: Susceptibility-weighted image
SNPSR: Swiss Neuropediatric Stroke Registry
TTTS: Twin-to-twin transfusion syndrome
UC: Umbilical cord
VUE: Villitis of unknown etiology
